# Early Pliocene onset of modern Nordic Seas circulation related to ocean gateway changes

**DOI:** 10.1038/ncomms9659

**Published:** 2015-10-28

**Authors:** Stijn De Schepper, Michael Schreck, Kristina Marie Beck, Jens Matthiessen, Kirsten Fahl, Gunn Mangerud

**Affiliations:** 1 Uni Research Climate, Bjerknes Centre for Climate Research, Allégaten 55, N-5007 Bergen, Norway; 2 Department of Earth Science, University of Bergen, Allégaten 41, N-5007 Bergen, Norway; 3 Arctic Research Centre, Korea Polar Research Institute, 26 Songdomirae-ro, Yeonsu-gu, 406-840 Incheon, Korea; 4 Alfred Wegener Institute, Helmholtz Centre for Polar and Marine Research, Am Alten Hafen 26, 27568 Bremerhaven, Germany

## Abstract

The globally warm climate of the early Pliocene gradually cooled from 4 million years ago, synchronous with decreasing atmospheric CO_2_ concentrations. In contrast, palaeoceanographic records indicate that the Nordic Seas cooled during the earliest Pliocene, before global cooling. However, a lack of knowledge regarding the precise timing of Nordic Seas cooling has limited our understanding of the governing mechanisms. Here, using marine palynology, we show that cooling in the Nordic Seas was coincident with the first trans-Arctic migration of cool-water Pacific mollusks around 4.5 million years ago, and followed by the development of a modern-like Nordic Seas surface circulation. Nordic Seas cooling precedes global cooling by 500,000 years; as such, we propose that reconfiguration of the Bering Strait and Central American Seaway triggered the development of a modern circulation in the Nordic Seas, which is essential for North Atlantic Deep Water formation and a precursor for more widespread Greenland glaciation in the late Pliocene.

## Abstract (web summary)

The cause and timing of early Pliocene cooling in the Nordic Seas remains uncertain. Here, the authors present palynological data from the Norwegian and Iceland Seas that demonstrate regional cooling and the development of modern surface circulation around 4.5 Ma, likely related to Bering Strait gateway changes.

## Graphical abstract

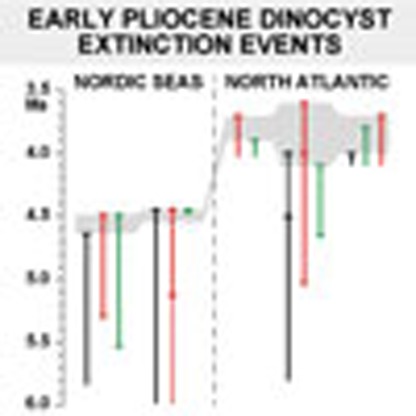

The early Pliocene (5–4 million years ago, Ma) is characterized by globally warmer sea surface temperatures and a reduced meridional temperature gradient compared with the present^1^,^2^,^3^ at atmospheric carbon dioxide concentrations of ∼400 p.p.m.^4^. Following the ‘early Pliocene climatic optimum' (4.4–4.0 Ma), atmospheric carbon dioxide concentrations decrease^4^ and a cooling is observed in sea surface temperature records from around the globe^2^. Local cooling of the seas around Iceland occurred earlier in the early Pliocene, together with^5^ or even before^6^ the first arrival of cool-water Pacific mollusks in Iceland. The arrival of cool-water Pacific mollusks^7^, which was recently re-dated to around 4.5 Ma (ref. 8), has been linked to inflow of Pacific waters into the Nordic Seas via the Bering Strait^7^,^8^,^9^.

Today, water flows from the Pacific through the narrow and shallow (∼50 m) Bering Strait into the Arctic and North Atlantic, influencing meridional overturning circulation strength and global climate^10^. The first inflow of Pacific water into the Arctic and Atlantic oceans has been determined based on the first occurrence of cool-water Pacific marine invertebrates (mollusks, gastropods) in 4.5-Myr-old sediments of the Tjörnes section in Iceland^8^,^11^. The implied northern migration pathway for these cool-water organisms is more plausible than a tropical pathway via the Central American Seaway. Although deep-water exchange across the Central American Seaway likely halted in the late Miocene^12^,^13^,^14^, a further shoaling phase in the early Pliocene has been interpreted between ∼4.8 and 4.2 Ma (refs 15, 16). Proxy data and modelling experiments suggest that the shoaling led to increased North Atlantic meridional overturning circulation^16^, a Nordic Seas circulation with a stronger Norwegian Atlantic Current and a cooler and fresher East Greenland Current^17^, but also the onset of northward flow through the Bering Strait into the Arctic^18^,^19^.

As the connection between the North Atlantic and Arctic Ocean, and a region of North Atlantic Deep Water formation, the Nordic Seas are an integral part of the Atlantic meridional overturning circulation^20^. The modern Nordic Seas can be divided into a polar, Arctic and Atlantic domain^21^ based on different surface water masses and the position of the polar and Arctic front (Fig. 1). The strong east–west sea surface temperature and salinity gradient is caused by inflow of relatively warm saline Atlantic water in the southeast, continuing as the Norwegian Atlantic Current towards the Arctic Ocean, and by export of cool, fresher Arctic water via the East Greenland Current in the west^22^. This Nordic Seas circulation was hypothesized to be initiated in the early Pliocene as a consequence of Central American Seaway closure^17^, but a precise timing, encompassing mechanism and conclusive data from the Nordic Seas remained elusive.

Here, using new marine palynological data (palynomorphs including dinoflagellate cysts and acritarchs) from Norwegian Sea Ocean Drilling Program (ODP) Hole 642B and an existing record from Iceland Sea ODP Hole 907A (ref. 23), we demonstrate that the Nordic Seas cooled and a zonal sea surface temperature gradient developed around 4.5 Ma, preceding the global climate cooling and atmospheric carbon dioxide decline from 4.0 Ma. We link this change in surface ocean conditions to inflow of fresher, cooler Pacific water via the Bering Strait into the Nordic Seas along the East Greenland Current pathway around 4.5 Ma.

## Results

### Marine palynology at Norwegian Sea ODP Hole 642B

In the early Pliocene, between 5.0 and 4.5 Ma, the palynological assemblages indicate an outer shelf environment influenced by warm, temperate waters. The dinoflagellate cyst assemblage contains the typical early Pliocene dinoflagellate cyst taxa *Batiacasphaera micropapillata* complex, *Corrudinium devernaliae*, *Operculodinium tegillatum*, *Pyxidinopsis vesiculata* and *Reticulatosphaera actinocoronata* (Fig. 2; Supplementary Data 1). The dominant dinoflagellate cyst taxa *B. micropapillata* complex (3–43%), *Spiniferites*/*Achomosphaera* spp. (5–42%), Protoperidinioids (6–54%) and occasionally *Nematosphaeropsis labyrinthus* (2–29%) indicate an outer shelf, consistent with the location of ODP Hole 642B on the Vøring Plateau. The acritarch assemblage is dominated by *Cymatiosphaera*? *invaginata*, and occasionally by *Lavradosphaera crista* and Cyst type I of de Vernal and Mudie (1989). The high acritarch (51,388±14,187 acritarchs per g sediment) and dinoflagellate cyst (32,993±9,140 cysts per g sediment) concentrations, and the presence, but not dominance, of several oceanic genera (for example, *Impagidinium*, *Invertocysta*) are consistent with a nutrient-rich outer shelf environment. Both dinoflagellate cysts with a modern (sub)tropical distribution (for example, *Melitasphaeridium choanophorum*, *Tuberculodinium vancampoae*) and with a cool-water affinity (for example, *Filisphaera filifera*) occur in this interval. The high Warm/Cold index (W/C index, see Methods), due to the higher number of warm-water taxa (mainly *Operculodinium israelianum*, *Operculodinium*? *eirikianum*, *O. tegillatum* and *M. choanophorum*), indicates warm temperate conditions. A brief cooling can be interpreted from the W/C index around 4.9–4.8 Ma (Fig. 3; Supplementary Data 1), due to an increase of the cool-water species *F. filifera*.

After 4.5 Ma, the palynological assemblages indicate an outer shelf environment influenced by cooler waters compared with the interval 5.0–4.5 Ma. The typical early Pliocene marker taxa *B. micropapillata* complex, *C. devernaliae*, *O. tegillatum*, *P. vesiculata*, *R. actinocoronata* and the acritarch Cyst type I of de Vernal and Mudie (1989) disappeared around 4.5 Ma (Fig. 2; Supplementary Data 1). The dominant taxa are cysts of *Protoceratium reticulatum* (5–45%), *Spiniferites*/*Achomosphaera* spp. (14–23%), *N. labyrinthus* (5–12%) and *F. filifera* (0–13%). The previously abundant Protoperidinioids decrease to less than 5% of the total assemblage, indicating reduced nutrient availability. Palynomorph concentrations drop considerably to 8,684±1,361 dinoflagellate cysts per g sediment and 22,803±3,148 acritarchs per g sediment. Such concentrations, the abundant *Spiniferites*/*Achomosphaera* spp. (14–21%) and dominant cysts of *P. reticulatum* (29–45%), which are known to be abundant around shelf edge environments^24^, are consistent with an outer shelf environment. The occurrence of several oceanic taxa (for example, *Impagidinium*) indicates influence of open-ocean waters. Cysts of *P. reticulatum*, an indicator for inflow of warm North Atlantic waters into the Pliocene Nordic Seas^25^,^26^, are present in the record since 4.5 Ma and become dominant from 4.23 Ma. The (sub)tropical and thermophilic dinoflagellate cysts *Lingulodinium machaerophorum*, *M. choanophorum*, *Dapsilidinium pseudocolligerum* and *O*.? *eirikianum* further confirm that warm Atlantic water influenced the Norwegian Sea. However, increasing numbers of cool-water taxa *F. filifera* and *Impagidinium pallidum* and a decrease of warm-water taxa (for example, *O*.? *eirikianum*, *O. tegillatum* and *O. israelianum*) results in a drop in the W/C index and thus a cooling relative to the period between 5.0 and 4.5 Ma.

### Marine palynology at Iceland Sea ODP Hole 907A

In the early Pliocene between 5.0 and 4.5 Ma, the palynological assemblages indicate an open-ocean environment, with input from the shelf, influenced by temperate waters. Comparable to the Norwegian Sea, the same early Pliocene marker species such as *O. tegillatum*, *O*.? *eirikianum*, *B. micropapillata* complex and *R. actinocoronata* occur in ODP Hole 907A (Fig. 2; Supplementary Data 2). The dinoflagellate cyst assemblage is dominated by *N. labyrinthus* (0–65%), and occasionally *B. micropapillata* complex (0–47%), *Spiniferites elongatus* (1–50%) and *Spiniferites* spp. (0–12%). Protoperidinioids (2–15%) are common to abundant throughout the interval. Acritarchs are also abundant and dominated by *C*.? *invaginata* and *L. crista*, the same taxa as in the Norwegian Sea. Together this suggests an open-ocean environment, with occasional influx from the outer shelf (*Spiniferites* spp.). This interval also records the highest concentrations of the entire studied interval, with dinoflagellate cyst concentrations of 9,073±1,308 cysts per g sediment, and acritarch concentrations of 6,199±958 acritarchs per g sediment. In general, the assemblage composition at ODP Hole 907A is comparable to ODP Hole 642B, but the more oceanic species *N. labyrinthus* is dominant and dinoflagellate cyst concentrations are lower. This is consistent with the more open-ocean setting of ODP Hole 907A compared with the outer shelf environment at the Vøring Plateau ODP Hole 642B. The co-occurrence throughout the interval of dinoflagellate cysts with a warm-water affinity such as *Impagidinium aculeatum*, *Invertocysta lacrymosa*, *M. choanophorum*, *O. israelianum*, *O. tegillatum* and *O*.? *eirikianum* and the cool-tolerant taxa *S. elongatus* (common to dominant) and *I. pallidum* (rare to common) indicates temperate conditions in the Iceland Sea and inflow of warm Atlantic water (see Supplementary Note 1).

After 4.5 Ma, the palynological record indicates an open-ocean environment, with cool-water conditions between 4.5 and 4.26 Ma and followed by a long barren interval until 2.6 Ma (with a few exceptions, see Supplementary Note 2), indicating harsh conditions for dinoflagellate cysts and acritarchs. The Pliocene marker species, including the warm-water species *O*.? *eirikianum*, and Protoperidiniods disappear between 4.5 and 4.4 Ma, but dinoflagellate cysts remain present until 4.26 Ma (Fig. 2; Supplementary Data 2). Nevertheless, dinoflagellate cyst and acritarch concentrations decrease considerably after 4.5 Ma leading to a total crash at 4.14 Ma, when the assemblage becomes taxonomically depleted. In contrast to the transition towards an assemblage dominated by cysts of *P. reticulatum* observed in ODP Hole 642B, the impoverished assemblage in ODP Hole 907A between 4.5 and 4.26 Ma remains dominated by the oceanic species *N. labyrinthus*, and occasionally *Impagidinium* cf. *pacificum* and *I. pallidum.* This indicates an open-ocean environment, with little or no influence from the shelf, and cool-water conditions. Warm-water taxa (for example, *O*.? *eirikianum*) that were recorded previously at this site are almost entirely absent after 4.45 Ma. From 4.26 to 2.6 Ma, ODP Hole 907A is characterized by a long interval of nearly barren palynological samples containing less than 10 cysts counted per sample and concentrations below 60 cysts per g sediment. The absence of cysts of *P. reticulatum* and thermophilic taxa, which respectively thrive and persist in the Norwegian Sea, indicates that warm Atlantic water did not influence the Iceland Sea.

### Iceland Sea ODP Hole 907A alkenone sea surface temperatures

Our low-resolution sea surface temperature reconstruction for Iceland Sea ODP Hole 907A based on the alkenone unsaturation index (
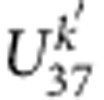
) indicates considerably higher annual sea surface temperatures in the early Pliocene compared with present (Fig. 3; Supplementary Table 1). At 4.5 Ma, a persistent cooling can be observed followed by a further cooling after 4 Ma, yet at all time remaining warmer than present.

## Discussion

If the late Miocene to earliest Pliocene (>4.5 Ma) Nordic Seas circulation were the same as today, a clear contrast between the warm Atlantic-influenced Norwegian Sea and the cool Arctic-influenced Iceland Sea should be visible in the palynological record. In contrast, our early Pliocene (5.0–4.5 Ma) dinoflagellate cyst records show that only a weak-to-absent zonal sea surface temperature gradient existed between the Norwegian and Iceland seas, and second, that the surface water evolution in both seas is coupled and influenced by Atlantic water. Notwithstanding some differences due to the different environment at both studied sites, the earliest Pliocene records from the Norwegian Sea and Iceland Sea^23^ are very comparable before 4.5 Ma: at both sites the same early Pliocene markers and abundant taxa (*Batiacasphaera*, *N. labyrinthus*, *O. tegillatum* and *Spiniferites*) occur and palynomorph concentrations reach the highest values for the late Miocene to late Pliocene interval (Fig. 2). The dinoflagellate cyst assemblages indicate warm temperate conditions in the Norwegian Sea and temperate conditions in the Iceland Sea, implying that a strong, modern zonal surface temperature gradient was absent (Fig. 3). The presence of (sub)tropical dinoflagellate cysts (for example, *L. machaerophorum*, *M. choanophorum* and *T. vancampoae*) in both the Norwegian Sea and Iceland Sea indicate that warm Atlantic water influenced the entire Nordic Seas. Furthermore, ostracod^5^, mollusk^7^,^27^ and dinoflagellate cyst^28^ assemblages as well as mollusk δ^18^O palaeotemperature reconstructions^6^ from the shallow marine Tjörnes Beds in northeastern Iceland indicate warm conditions at that time (Fig. 3). Also our low-resolution alkenone sea surface temperature record from the Iceland Sea indicates warm surface water conditions before 4.5 Ma and a variability that is largely comparable to the Norwegian Sea W/C index (Fig. 3; Supplementary Note 3). This suggests a coupling of the temperature and surface water masses evolution in the Norwegian Sea and Iceland Sea between 5.0 and 4.5 Ma (Fig. 4).

The Norwegian Sea and Iceland Sea temperature evolution becomes uncoupled after 4.5 Ma and the Atlantic water influence weakened in the Iceland Sea, which became gradually more influenced by cool, fresh water from the East Greenland Current. Around 4.5 Ma, the Norwegian Sea and Iceland Sea dinoflagellate cyst assemblage underwent a major turnover (Fig. 2; Supplementary Table 2), which we interpret as a cooling followed by the development towards the modern circulation in the Nordic Seas. In Norwegian Sea ODP Hole 642B, this turnover is expressed in a cluster of extinction events, a decrease in the W/C index and a shift from assemblages with an important heterotrophic component (*Barssidinium*, *Brigantedinium*, *Selenopemphix*, round brown cysts) to assemblages dominated by cysts of *P. reticulatum* (Fig. 2). The distribution of the cysts of *P. reticulatum* in recent sediments closely matches the Gulf Stream and North Atlantic Drift in the North Atlantic^29^ and the Norwegian Atlantic Current in the Nordic Seas^30^. In Pliocene records, the dominant presence of this species has been interpreted as evidence for the North Atlantic Current in the eastern North Atlantic^31^ and inflow of North Atlantic water into the Nordic Seas^25^. Its appearance in Norwegian Sea ODP Hole 642B after 4.5 Ma onwards is consistent with the first establishment of a more modern-like Norwegian Atlantic Current. A reduction of warm-water species and increase of cooler-water species (for example, *Habibacysta tectata* and *F. filifera*) after 4.5 Ma is reflected in a decreasing W/C index, demonstrating a significant early Pliocene cooling (Fig. 3). Nevertheless, (sub)tropical and thermophilic species such as *O*.? *eirikianum*, *M. choanophorum* and *D. pseudocolligerum* continue to be present, suggesting that warmer-than-present sea surface conditions persisted in the Norwegian Sea.

In contrast, cysts of *P. reticulatum* were never recorded in the Iceland Sea, and *M. choanophorum* and *O*.? *eirikianum* disappear at 4.5 Ma, indicating substantial cooling and waning of an Atlantic water influence there. In fact, the palynomorph assemblages and alkenone record in ODP Hole 907A provide evidence that a cool Arctic domain and proto-East Greenland Current were established after 4.5 Ma, when the early Pliocene productive interval is abruptly halted and followed by a long barren interval. At 4.5 Ma, the same dinoflagellate cyst species as in the Norwegian Sea go extinct, palynomorph concentrations and productivity decrease and heterotrophic species disappear (Fig. 2). This turnover and the following barren interval have been attributed to decreasing sea surface temperatures and salinity changes due to arrival of cool, fresh waters of a proto-East Greenland Current at the site^23^. A cooling of the Iceland Sea after 4.5 Ma is evident in our low-resolution ODP Hole 907A alkenone sea surface temperature record, as well as in ostracod and mollusk-isotope palaeotemperature reconstructions from the Tjörnes Beds^5^,^6^ (Fig. 3). The further cooling of sea surface temperatures in the Iceland Sea and expansion of a sea-ice cover from the Central Arctic Ocean to the marginal eastern Arctic^32^ after 4.0 Ma provide evidence for increasingly cool and fresh water transport via the East Greenland Current. The contemporaneous onset of biogenic opal accumulation at ODP Site 646 in the Labrador Sea was similarly linked to sea-ice-edge diatom production and significant surface water cooling due to export of polar water^17^. But probably the best evidence for Pacific cool-water inflow into the Nordic Seas around 4.5 Ma is the first occurrence of cool-water Pacific mollusks (for example, *Mya arenaria* Linnaeus, *Macoma obliqua* Sowerby) in sediments of the Tjörnes section in Iceland and even in the North Sea Basin (see Supplementary Table 3).

The cooling and freshening of the East Greenland Current and development of an Arctic domain in the Nordic Seas can be directly tied to the inflow of fresher Pacific water via the Bering Strait (Fig. 4). First, for Pacific mollusks to have arrived in the North Atlantic realm around 4.5 Ma, the Arctic Ocean must have been (seasonally) sea-ice free and Nordic Seas surface water temperatures must have decreased to subarctic values. Before 4 Ma, the seas along the Arctic Ocean margins were perennially ice-free^32^, and from 4.5 Ma Nordic Seas surface water temperatures dropped considerably. This is evidenced in the palynomorph assemblage turnover at both the studied sites, the decrease in the W/C index at ODP Hole 642B, the cooling recorded in the Iceland Sea alkenone record, palaeotemperature reconstructions at the Tjörnes Beds^5^,^6^ and invasion of cool-water mollusks (Fig. 3). Second, and maybe most importantly, for Pacific mollusks to have arrived in the Tjörnes section around 4.5 Ma, crucially the Pacific–Arctic–Atlantic gateways must have been open. One of the hypotheses explaining the strongly asymmetric trans-Arctic interchange of marine invertebrates—Pacific invaders outnumber those from the Arctic–Atlantic by a ratio of 8:1—is a dominantly northward flow of Pacific water through an open Bering Strait into the Arctic and eventually Nordic Seas^7^,^33^. The condition of open gateways was established by the late Miocene, when the Fram Strait was open for deep-water exchange^26^ and also the Bering Strait was open by 5.5–5.4 Ma (refs 34, 35), possibly as early as 7.4–7.3 Ma (ref. 36). The Bering Strait and Fram Strait were then the only high-latitude connections between the Atlantic and Pacific, since the Canadian Arctic Archipelago was closed and Barents Sea subaerially exposed^37^. Our data are consistent with northward flow of cool, fresh Pacific water through the Bering Strait continuing via the Arctic into the Nordic Seas along the East Greenland Current pathway. In the tropics, deep-water exchange across the Central American Seaway was likely halted by the late Miocene^12^,^13^,^14^, but shallow exchange became gradually more restricted between 4.8–4.2 Ma (refs 15, 16). Such exchange persisted until ∼2.5 Ma (ref. 12), possibly via transient channels west of the Panama Canal Basin^14^, allowing an active North Atlantic meridional overturning circulation. Early Pliocene Central American Seaway shoaling has been linked mainly to changes in the North Atlantic surface and deep circulation^12^,^15^,^16^,^17^, but it may also have led to Pacific-to-Atlantic flow through the Bering Strait^9^ via an increased North Pacific sea level relative to the North Atlantic^18^,^19^. Today, water flow through the open Bering Strait is mainly from the Pacific to the Arctic and is sustained by a sea-level difference and salinity contrast between both basins^38^. Therefore, supported by a contemporaneous timing of the changes at the Pacific–Atlantic gateways and the development towards a proto-East Greenland Current and Norwegian Atlantic Current after 4.5 Ma, there thus is a link between northward flow through the Bering Strait—itself probably due to Central American Seaway shoaling—and the development of the modern Nordic Seas circulation. It is important to note that the flow through the Bering Strait may have had a more direct impact on Nordic Seas circulation, deep-water formation and global climate than previously believed.

The dinoflagellate cysts that disappear from the Nordic Seas around 4.5 Ma only go extinct in the North Atlantic after ∼4 Ma, indicating a 500-kyr delay (Fig. 2). The North Atlantic extinctions are coeval with a decreasing global sea surface temperature^2^, implying that the development of the zonal sea surface temperature gradient in the Nordic Seas around 4.5 Ma predates the early Pliocene global cooling. Therefore, the fundamental oceanographic change in the Nordic Seas was not caused by global climate evolution^2^, but is more likely attributable to changes in Pacific–Atlantic oceanic gateway configurations.

It is interesting that North Atlantic surface waters cooled from 4 Ma onwards, whereas changes in Nordic Seas circulation and North Atlantic deep-water circulation are apparent 500-kyrs earlier (Figs 2 and 3). In the early Pliocene northeast Atlantic, a major unconformity was related to changed deep-ocean circulation^39^. At the Eirik Drift, an excellent recorder of deep-water currents originating from overflows from Nordic Sea Deep Water formation regions, an erosional unconformity and a high-amplitude reflector have been identified at 4.5 Ma (ref. 40). This reflector indicates the influence of a strong deep current likely due to circulation changes and enhanced deep-water formation in the Nordic Seas. Indeed, the Denmark Strait overflow increased around that time^17^, possibly aided by regional subsidence, with sustained production of Northern Component Water thereafter^41^. On the Gloria Drift, sediment accumulation started at 4.5 Ma, and several North Atlantic sediment drifts reveal overall highest apparent sedimentation rates in the early Pliocene^42^, indicating a major change towards a bottom-current-dominated regime. The early Pliocene change in North Atlantic deep-water circulation is simultaneous with development of the modern Nordic Seas circulation at 4.5 Ma underlining the importance of Pacific water flow into the Atlantic via the Bering Strait on the thermohaline circulation.

In summary, a modern Nordic Seas circulation with a strong east–west surface water temperature gradient developed since 4.5 Ma as a result of contemporaneous gateway reconfigurations in the Central American Seaway and Bering Strait. The flow of Pacific waters through the Bering Strait had a direct effect on Nordic Seas circulation, but may ultimately be caused by impeded flow through the Central American Seaway and consequent buildup of a Pacific–Atlantic salinity and sea-level gradient. The Pacific-to-Atlantic flow via the Bering Strait cooled and freshened the East Greenland Current, in fact establishing an Arctic domain in the Nordic Seas and isolating Greenland from warm Atlantic waters of the Norwegian Atlantic Current. This was an important precondition for more widespread glaciation on Greenland that remained restricted in the early Pliocene^43^, but gradually increased following regional circum-Arctic uplift around 4.0 Ma (ref. 26) and late Pliocene decreasing atmospheric carbon dioxide concentrations^4^. Establishment of a modern Nordic Seas circulation pattern also intensified North Atlantic Deep Water formation, making the region a fundamental part of the Atlantic meridional overturning circulation and a key region in global climate variability since the early Pliocene.

## Methods

### Marine palynology

**Figure 1:**
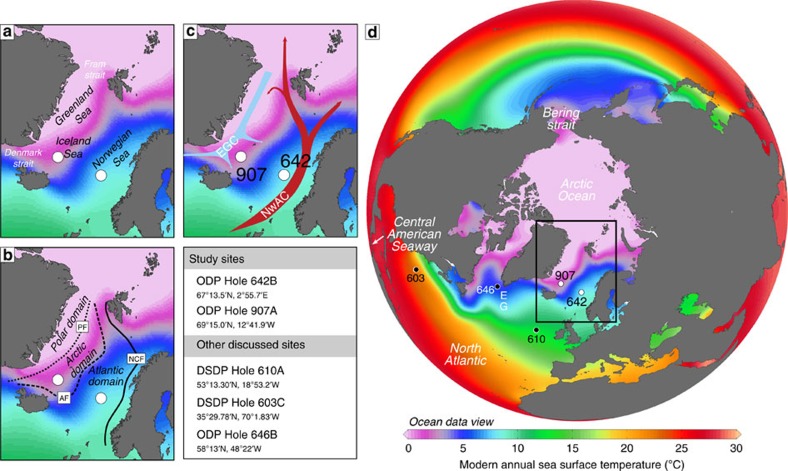
Modern Nordic Seas oceanography. (**a**) The Nordic Seas encompass the Norwegian Sea, Iceland Sea and Greenland Sea. (**b**) The Nordic Seas divide into a polar, Arctic and Atlantic domain^21^ based on the position of the Norwegian Continental Shelf front (NCF), Arctic front (AF) and polar front (PF). (**c**) Location of ODP Sites 642 and 907, and the modern surface circulation with inflow of warm Atlantic water via the Norwegian Atlantic Current (NwAC) and outflow of cool, polar water via the East Greenland Current (EGC). (**d**) Map of the Northern Hemisphere modern annual sea surface temperatures (from the World Ocean Atlas 2005, ref. 52), showing all ocean drill sites mentioned in the text. E, Eirik Drift; G, Gloria Drift. Black box shows study area in the Nordic Seas (see a,b,c).

**Figure 2:**
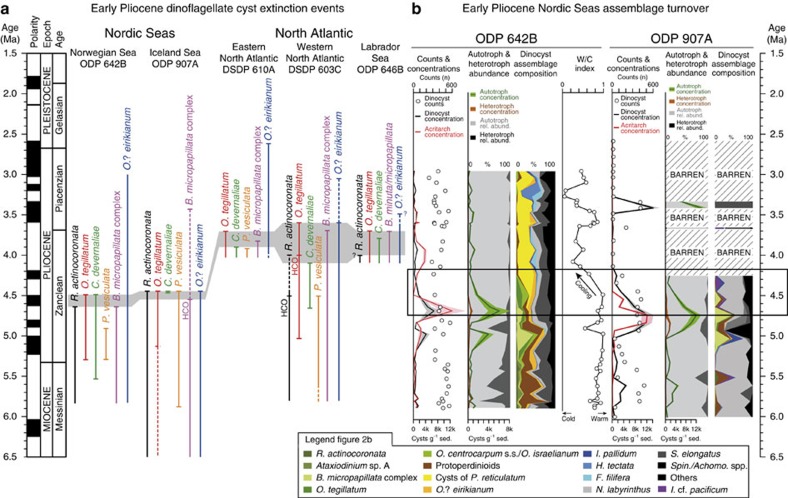
Dinoflagellate cyst biostratigraphic and palaeoenvironmental reconstructions for the early Pliocene Nordic Seas. (**a**) Early Pliocene dinoflagellate cyst extinction events at ocean drill sites in the Nordic Seas and North Atlantic demonstrating the synchronous response of the dinoflagellate cyst assemblage around 4.5 Ma within the Nordic Seas, and the delayed extinctions in the North Atlantic. The grey-shaded area indicates the time interval wherein *R. actinocoronata*, *O. tegillatum*, *C. devernaliae* and *B. micropapillata* complex get extinct. HCO, highest common occurrence. (**b**) Dinoflagellate cysts and acritarch concentrations and assemblage composition reflect a major assemblage turnover around 4.5 Ma (as indicated by the black rectangle) at ODP Hole 642B and ODP Hole 907A. The Warm/Cold (W/C) index at ODP Hole 642B reveals a cooling associated with the assemblage turnover. Shaded areas for concentration records indicate error. Barren intervals contain less than 10 counted dinoflagellate cysts. Inset box legend Fig. 2b presents the colour coding used for the dinoflagellate cyst assemblage composition graphs. Spin./Achomo. spp., *Spiniferites*/*Achomosphaera* spp.

**Figure 3:**
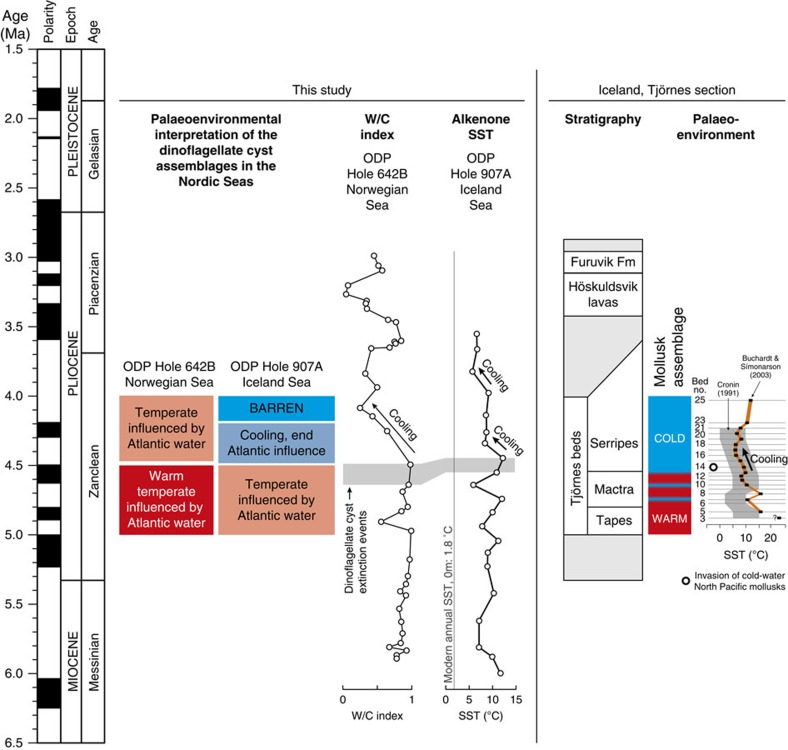
Evidence for cooling in the Nordic Seas region around 4.5 Ma. The Nordic Seas dinoflagellate cyst assemblages, the Norwegian Sea ODP Hole 642B W/C index, the Iceland Sea ODP Hole 907A low-resolution alkenone sea surface temperature (SST), and the mollusk assemblages, marine ostracod palaeotempreature estimates^5^ (grey shading) and mollusk δ^18^O palaeotemperature estimates^6^ (orange curve) from shallow marine deposits in the Tjörnes section (NE Iceland) all show distinct cooling around 4.5 Ma. Tjörnes Beds stratigraphy and bed numbering (horizontal lines indicate approximate position of beds) are from the age model of ref. 8. Note that position and age of the beds are approximate, but beds 11–14 belong to the C3n.2n subchron (normal polarity, 4.631–4.493 Ma) and bed 25 is in the C2Ar subchron (reversed polarity, 4.187–3.596 Ma)^8^. Modern annual average sea surface temperatures from World Ocean Atlas (2005)^52^.

**Figure 4:**
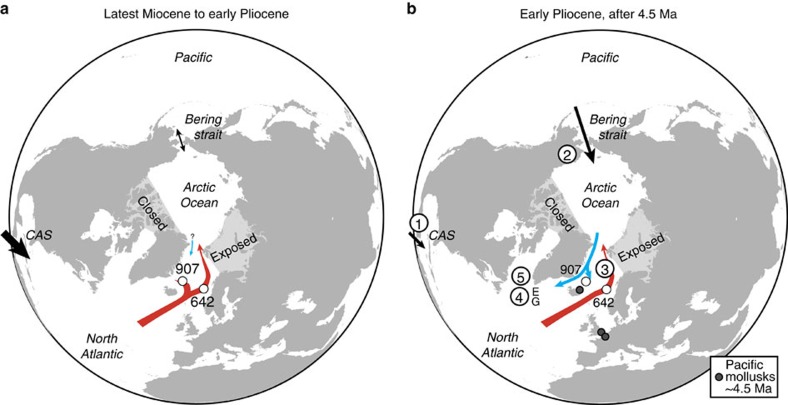
Latest Miocene and early Pliocene Nordic Seas surface circulation in relation to Pacific–Arctic–Atlantic gateway evolution. (**a**) Before 4.5 Ma, the Nordic Seas were mainly influenced by warm North Atlantic waters. (**b**) After 4.5 Ma, shoaling at Central American Seaway and reduction in Pacific–Atlantic exchange (1), and opening and northward flow through the Bering Strait (2) led to development of a modern-like Nordic Seas circulation with an East Greenland Current and Norwegian Atlantic Current (3), changes in deep-water circulation at the Eirik (E) and Gloria (G) Drift deposits (4) and cooling in the Labrador Sea (5).

We investigated the dinoflagellate cysts and acritarchs of 40 samples across the time interval between 5.8 and 3.0 Ma (94.95–64.54 mbsf) at Vøring Plateau ODP Hole 642B (67°13.5′N, 2°55.7′E; water depth 1,268 m; Fig. 1). The samples were prepared using a standard palynological maceration procedure, which included weighing dried sediment, adding one *Lycopodium clavatum* tablet (batch #483216), digestion cycles in cold HF and cold HCl, no oxidation, occasional mild ultrasonic treatment and sieving at 10 μm before mounting the residue on microscope slides. Slides were then counted using a light microscope at × 400 magnification along non-overlapping traverses until at least 250 specimens were counted, or until the slide was completely scanned.

The total error on the palynomorph concentration is calculated following the method outlined in ref. 44: total error 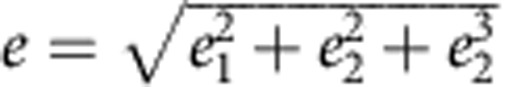 with e_1_=error on number of spores in *L. clavatum* marker tablets, e_2_=error on number of dinoflagellate cysts counted and e_3_=error on number of *L. clavatum* markers counted.

The Warm/Cold (W/C) index is calculated based on the dinoflagellate cyst occurrences according to the formula W/C=*n*W/(*n*W+*n*C), with *n*=number of specimens counted, W=warm-water species, C=cold-water species. The ecological preference of each species included in the index was considered in relation to their distribution in the modern oceans^45^,^46^ and in Pliocene deposits^47^,^48^,^49^,^50^. This resulted in using the following species as warm-water species: *Achomosphaera andalousiensis* subsp. *suttonensis*, *D. pseudocolligerum*, *Hystrichokolpoma rigaudiae*, *I. aculeatum*, *I. paradoxum*, *I. patulum*, *I. solidum*, *I. lacrymosa*, *I. tabulata*, *L. machaerophorum*, *M. choanophorum*, *O*.? *eirikianum*, *O. centrocarpum* s.s.*/O. israelianum*, *O. tegillatum*, *S. mirabilis/hyperacanthus*, *Tectatodinium pellitum* and *Tubercoludinium vancampoae.* The cool-water species were: *Bitectatodinium tepikiense*, *F. filifera*, *H. tectata*, *I. pallidum* and *S. elongatus.* A reliable W/C index could not be calculated for ODP Hole 907A over the entire studied interval, because the total dinoflagellate cyst count was often lower than 100 cysts per sample in the Pliocene and/or the sum of cold and warm specimens was lower than 10.

A full authorial citation of the dinoflagellate cyst species mentioned in the text and figures is given in the Supplementary Note 4.

All raw data from ODP Hole 642B is available at doi: 10.1594/PANGAEA.846838; data from ODP Hole 907A (69°15.0′N, 12°41.9′W; water depth 2,036 m; Fig. 1) is available at doi:10.1594/PANGAEA.807163. Summarized data is presented in Supplementary Data 1 and 2.

### Biomarker analyses

For alkenone analyses, freeze-dried and homogenized sediments (2 to 4 g) were extracted with an Accelerated Solvent Extractor (DIONEX, ASE 200; 100 °C, 1,000 psi, 5 min) using dichloromethane and methanol (99:1, v/v) as solvent. The separation of compounds was carried out by open-column chromatography (SiO_2_) using *n*-hexane and dichloromethane (1:1, v/v), and dichloromethane. The composition of alkenones was analysed with an Agilent gas chromatograph (7890, column 60 m × 0.32 mm; film thickness 0.25 μm; liquid phase: DB1-MS) using a temperature program as follows: 60 °C (3 min), 150 °C (rate: 20 °C min^−1^), 320 °C (rate: 6 °C min^−1^) and 320 °C (40 min isothermal). For splitless injection, a cold injection system was used (60 °C (6 s), 340 °C (rate: 12 °C s^−1^), 340 °C (1 min, isothermal)). Helium was used as carrier gas (1.2 ml min^−1^). Individual alkenone (C_37:3_, C_37:2_) identification is based on retention time and the comparison with an external standard.

The alkenone unsaturation index (
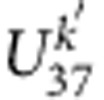
) as proxy for mean annual sea surface temperature (°C) was calculated following ref. 51, based on a global core-top calibration (60°N–60°S): 
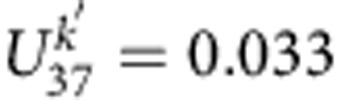
 × T (°C)+0.044. The s.e. of the calibration is reported as ±0.050 
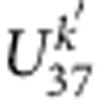
 units or ±1.5 °C. The instrument stability was continuously controlled by re-runs of an external alkenone standard (extracted from *Emiliania huxleyi* cultures with known growth temperature) during the analytical sequences. The range of the total analytical error calculated by replicate analyses is less than 0.4 °C. The data are presented in Supplementary Table 1 and are available from doi:10.1594/PANGAEA.848671.

### Age models

Tie points used to construct the age model of ODP Holes 642B, 646B, 907A and Deep Sea Drilling Project (DSDP) Holes 603C, 610A are presented in the Supplementary Tables 4–8.

## Additional information

**How to cite this article:** De Schepper, S. *et al*. Early Pliocene onset of modern Nordic Seas circulation related to ocean gateway changes. *Nat. Commun.* 6:8659 doi: 10.1038/ncomms9659 (2015).

## Supplementary Material

Supplementary Information Supplementary Tables 1-8, Supplementary Notes 1-4 and Supplementary References.

Supplementary Data 1 Relative and absolute abundance data for dinoflagellate cysts and acritarchs, and the calculated W/C index of ODP Hole 642B.

Supplementary Data 2 Relative and absolute abundance data for dinoflagellate cysts and acritarchs of ODP Hole 907A.
