# Supplementary material for: Early Pliocene onset of modern Nordic Seas circulation related to ocean gateway changes
Source: Nat Commun. 2015 Oct 28;6:8659. doi: 10.1038/ncomms9659 (PMC4639897; doi:10.1038/ncomms9659)
Supplement: Supplementary Information — Supplementary Tables 1-8, Supplementary Notes 1-4 and Supplementary References. [file ncomms9659-s1.pdf]

SUPPLEMENTARY TABLE 1

Latest Miocene to early Pliocene alkenone sea surface temperatures (annual) for ODP Hole 907A.

**Site, Hole**	**Core-section**	**Depth in section** (cm)	**Depth** (mbsf)	**Age** (Ma)	$U^{k'}_{37}$	**SST** (°C)
907A	8H2	40–42	66.2	3.55	0.261	**6.57**
907A	8H2	140–142	67.2	3.66	0.264	**6.66**
907A	8H3	90–92	68.2	3.82	0.203	**4.82**
907A	8H4	43–45	69.2	3.98	0.346	**9.16**
907A	8H4	140–142	70.2	4.14	0.326	**8.56**
907A	8H5	90–92	71.2	4.26	0.332	**8.72**
907A	8H6	32–34	72.1	4.34	0.301	**7.80**
907A	8H6	140–142	73.2	4.45	0.447	**12.23**
907A	9H1	40–42	74.2	4.55	0.404	**10.90**
907A	9H1	140–142	75.2	4.64	0.223	**5.42**
907A	9H2	90–92	76.2	4.74	0.415	**11.23**
907A	9H3	41–43	77.2	4.84	0.350	**9.26**
907A	9H3	140–142	78.2	4.94	0.299	**7.72**
907A	9H4	93–95	79.2	5.04	0.415	**11.25**
907A	9H5	40–42	80.2	5.13	0.339	**8.95**
907A	9H5	140–142	81.2	5.23	0.337	**8.88**
907A	9H7	40–42	83.2	5.42	0.359	**9.54**
907A	10H2	40–42	85.2	5.62	0.277	**7.06**
907A	10H2	140–142	86.2	5.71	0.438	**11.94**
907A	10H3	90–92	87.2	5.81	0.275	**7.1**
907A	10H4	17.5–19.5	88.0	5.88	0.372	**9.94**
907A	10H4	140–142	89.2	6.00	0.427	**11.61**

SUPPLEMENTARY TABLE 2

Age estimates for dinoflagellate cyst extinctions recorded in the early Pliocene. Ages are for highest occurrence (HO), unless specified otherwise. Highest common occurrence = HCO; highest persistent occurrence = HPO. All raw data is available from the indicated references.

**Hole**	**ODP Hole 642C**	**ODP Hole 907A**	**DSDP Hole 610A**	**ODP Hole 646B**	**ODP Hole 603C**
**Region**	**Norwegian Sea**	**Iceland Sea**	**E. North Atlantic**	**Labrador Sea**	**W. North Atlantic**
References	This study	Refs. 1,2	Refs. 3,4	Refs. 2,5,6	Refs. 2,7,8
Batiacasphaera micropapillata complex	4.64 Ma	4.55 Ma (HCO)	3.83 Ma as *B. minuta*	c. 3.7 Ma as *B. sphaerica*	3.7 Ma as *B. micropapillata* (microreticulate)
Corrudinium devernaliae	4.49 Ma	4.45 Ma	3.90 Ma	c. 3.8 Ma as *Corrudinium* sp. 1	4.1 Ma
Operculodinium? *eirikianum*	< 3.0 Ma	4.45 Ma	2.62 Ma	c. 3.5 Ma as *Operculodinium longispinigerum*	3.1 Ma (HO) 3.6 Ma (HPO)
Operculodinium tegillatum	4.49 Ma	4.45 Ma	3.71 Ma	c. 3.7 Ma as *O. crassum*	3.6 Ma (HO) 4.0 Ma (HCO)
Pyxidinopsis vesiculata	4.91 Ma	4.45 Ma	3.92 Ma	Not recorded	4.5 Ma
Reticulatosphaera actinocoronata	4.64 Ma	4.45 Ma	> 4.0 Ma (base of core)	c. 4.0 Ma as *Impletosphaeridium* sp.	4.0 Ma (HO) 4.5 Ma (HCO)

SUPPLEMENTARY TABLE 3

Ages of deposits in the North Sea and Nordic Seas containing cool-water mollusk species with a Pacific origin.

Location	Stratigraphic unit	Age (Ma)	Age reference	Mollusks reference
**Belgium**	Luchtbal Sands member (Oorderen Sands Formation)	4.2–3.7	9	10
	Kattendijk Sands Formation	4.7–4.4	9	11
**Eastern England**	Coralline Crag	4.4–3.8	9,12	10,13
**The Netherlands**	Lower Mollusk Zone D1 Oosterhout Formation	Early Pliocene	10,14	10
**Iceland**	*Serripes* Zone, Tjörnes Beds	4.5–4.0 Ma	15	16

SUPPLEMENTARY TABLE 4

Age model and stratigraphic events of Ocean Drilling Program (ODP) Hole 642B. Tie-points (in bold) used to construct the age model of ODP Hole 642B are based on the palaeomagnetic reversals of ref. 17, which have been updated to the Geological Time Scale 2012 (GTS2012)[18]. In this study, we identify in ODP Hole 642B the stratigraphic events of *B. micropapillata* complex (HO at 77.80 mbsf), *C. devernaliae* (HO at 77.35 mbsf), *O.? eirikianum* (ranges up to the top of studied interval), *O. tegillatum* (HO at 77.35 mbsf), *P. vesiculata* (HO at 82.01 mbsf) and *R. actinocoronata* (HO at 77.80 mbsf).

**Chron/ Subchron** Ref. 17	**Chron/Subchron** (GTS 2012)	**Chron boundary name**	**Upper boundary**		**Lower boundary**		**Mid-depth** (mbsf)	**Age** (Ma) ref. 17	**Age** (Ma) updated to GTS 2012
			**Sample**	**Depth** (mbsf)	**Sample**	Depth (mbsf)			
C2AN-2/C2AR-2	C2An.2n/ C2An.2r	Top Mammoth	9H-1, 41	66.81	9H-1, 71	67.11	66.96	3.08	3.207
C2AR-2/C2AN-3	C2An.2r / C2An.3n	Base Mammoth	9H-2, 11	68.01	9H-2, 41	68.31	68.16	3.18	3.330
C2AN-3/C2AR-3	C2An.3n/C2Ar	Top Gilbert	9H-3, 47	69.87	9H-3,71	70.11	69.99	3.40	3.596
C2AR-3/C3N-1	C2Ar/C3n.1n	Top Cochiti	9H-7, 56	75.96	10H-1, 9	75.99	75.98	3.88	4.187
C3N-1/C3R-1	C3n.1n/C3n.1r	Base Cochiti	10H-1, 70	76.60	10H-1, 101	76.91	76.76	3.97	4.300
C3R-1/C3N-2	C3n.1r/C3n.2n	Top Nunivak	10H-1, 130	77.20	10H-2, 11	77.51	77.36	4.10	4.493
C3N-2/C3R-2	C3n.2n/C3n.2r	Base Nunivak	10H-2, 11	77.51	10H-2, 41	77.81	77.66	4.24	4.631
C3R-2/C3N-3	C3n.2r/C3n.3n	Top Sidufjall	10H-3, 71	79.61	10H-3, 100	79.90	79.76	4.40	4.799
C3N-3/C3R-3	C3n.3n/C3N.3r	Base Sidufjall	10H-4, 126	81.66	10H-5, 11	82.01	81.84	4.47	4.896
C3R-3/C3N-4	C3N.3r/C3n.4n	Top Thvera	10H-5, 130	83.20	10H-6, 10	83.5	83.35	4.57	4.997
C3N-4/C3R-4(?)	C3n.4n/C3r	Base Thvera	10H-6, 71	84.11	10H-6, 100	84.4	84.255	4.77	5.235
C3R-4(?)/(C3A) C3AN-1	C3R/C3An.1n	Top Gilbert	12H-2, 71	97.11	12H-2, 101	97.41	97.26	5.35	6.033

SUPPLEMENTARY TABLE 5

Age model and stratigraphic events of Ocean Drilling Program (ODP) Hole 907A. Tie-points (in bold) used to construct the age model of ODP Hole 907A are based on the palaeomagnetic reversals of ref. 19. These reversals were updated to the Astronomically Tuned Neogene Time Scale (ATNTS) 2004[20] in ref. 2, and to the Geological Time Scale 2012[18] in this study. The ATNTS2004 and GTS2012 are identical back to 8.3 Ma[18]. Stratigraphic events in ODP Hole 907A of *C. devernaliae*, *O.? eirikianum*, *O. tegillatum*, *P. vesiculata*, *R. actinocoronata* (all HO at 73.2 mbsf) and *B. micropapillata* complex (HCO at 74.2 mbsf) are presented in ref. 2.

**Chron boundary name**	**Chron/Subchron**	**Depth** (mbsf)	**Depth** (mcd)	**Age** (Ma)	**Age** (Ma)	**Age** (Ma) updated to GTS2012
Top Mammoth	C2An.2n/ C2An.2r	**61.20**	67.93	3.22	3.207	**3.207**
Top Gilbert	C2An.2r / C2An.3n	**66.80**	74.54	3.58	3.596	**3.596**
Top Cochiti	C2An.3n/C2Ar	**70.50**	78.24	4.18	4.187	**4.187**
Base Gilbert	C2Ar/C3n.1n	**89.50**	97.64	5.89	6.033	**6.033**
Base 3An.1n	C3n.1n/C3n.1r	**92.25**	100.39	6.14	6.252	**6.252**
Top 3An.2n	C3n.1r/C3n.2n	**93.30**	101.72	6.27	6.436	**6.436**
Top 3Ar	C3n.2n/C3n.2r	**96.40**	104.82	6.57	6.733	**6.733**

SUPPLEMENTARY TABLE 6

Age model and stratigraphic events of Deep Sea Drilling Project (DSDP) Hole 603C. The tie-points based on the palaeomagnetic reversals[8] used to construct the age model for DSDP Hole 603C in ref. 21 are repeated here. The ages of all reversals are reported according to the Geological Time Scale 2012[18]. Stratigraphic events in DSDP Hole 603C of *B. micropapillata* complex (HO at 185.96 mbsf), *C. devernaliae* (HO at 215.68 mbsf), *O.*? *eirikianum* (HO at 123.08 mbsf, HPO at 172.77 mbsf), *O. tegillatum* (HO at 172.77 mbsf, HPO at 204.82 mbsf), *P. vesiculat*a (HO at 249.07 mbsf) and *R. actinocoronata* (HO at 208.16, HCO at 249.07 mbsf) are listed in Appendix A of ref. 2.

**Chron boundary name**	**Chron/Subchron**	**Depth** (mbsf)	**Age** (Ma)
Top Olduvai	C1r.3r/C2n	13.00	1.778
Base Olduvai	C2n/C2r.1r	45.00	1.945
Top Gauss	C2r.2r/C2An.1n	82.00	2.581
Top Kaena	C2An.1n/C2An.1r	116.00	3.032
Base Kaena	C2An.1r/C2An.2n	128.00	3.116
Top Mammoth	C2An.2n/C2An.2r	136.00	3.207
Base Mammoth	C2An.2r/C2An.3n	144.00	3.330
Top Gilbert	C2An.3n/C2Ar	174.00	3.596
Top Cochiti	C2Ar/C3n.1n	222.00	4.187
Base Cochiti	C3n.1n/C3n.1r	232.00	4.300
Top Nunivak	C3n.1r/C3n.2n	244.00	4.493
Top Sidufjall	C3n.2n/C3n.3r	277.00	4.799
Base Thvera	C3n.4n/C3r	314.00	5.235

SUPPLEMENTARY TABLE 7

Age model and stratigraphic events of Deep Sea Drilling Project (DSDP) Hole 610A. The tie-points based on the palaeomagnetic reversals[22], benthic isotope stratigraphy[23] and one calcareous nannofossil extinction event used to construct the age model for DSDP Hole 610A in ref. 4 are repeated here. The ages are reported according to the Geological Time Scale 2012[18]. Stratigraphic events in DSDP Hole 610A of *B. micropapillata* complex (HO at 186.49 mbsf), *C. devernaliae* (HO at 191.67 mbsf), *O.? eirikianum* (HO at 128.47 mbsf), *O. tegillatum* (HO at 177.35 mbsf) and *P. vesiculata* (HO at 193.28 mbsf) are listed in refs. 3 and 4.

**Stratigraphic event**	**Chron/Subchron**	**Depth** (mbsf)	**Age** (Ma)
Top Matuyama	C1n/C1r.1r	42.46	0.781
Top Jaramillo	C1r.1r/C1r.1n	53.57	0.988
Base Jaramillo	C1r.1n/C1r.2n	56.93	1.072
Top Olduvai	C1r.3r/C2n	93.48	1.778
Base Olduvai	C2n/C2r.1r	98.63	1.945
Top Réunion	C2r.1r/C2r.1n	106.72	2.128
Base Réunion	C2r.1n/C2r.2r	108.37	2.148
$\delta^{18}O$ stratigraphy		120.76	2.440
$\delta^{18}O$ stratigraphy		126.15	2.560
Top Gauss	C2r.2r/C2An.1n	127.35	2.581
$\delta^{18}O$ stratigraphy		128.69	2.623
$\delta^{18}O$ stratigraphy		133.95	2.723
$\delta^{18}O$ stratigraphy		138.60	2.805
Top Kaena	C2An.1n/C2An.1r	150.89	3.032
Base Kaena	C2An.1r/C2An.2n	154.66	3.116
$\delta^{18}O$ stratigraphy		155.26	3.135
Top Mammoth	C2An.2n/C2An.2r	157.06	3.207
$\delta^{18}O$ stratigraphy		157.67	3.215
$\delta^{18}O$ stratigraphy		158.32	3.220
$\delta^{18}O$ stratigraphy		159.32	3.230
$\delta^{18}O$ stratigraphy		159.60	3.235
$\delta^{18}O$ stratigraphy		159.79	3.246
$\delta^{18}O$ stratigraphy		159.91	3.255
$\delta^{18}O$ stratigraphy		160.06	3.260
$\delta^{18}O$ stratigraphy		160.29	3.265
$\delta^{18}O$ stratigraphy		160.36	3.270
$\delta^{18}O$ stratigraphy		160.96	3.280
$\delta^{18}O$ stratigraphy		161.10	3.285
$\delta^{18}O$ stratigraphy		161.43	3.294
$\delta^{18}O$ stratigraphy		161.71	3.300
$\delta^{18}O$ stratigraphy		162.91	3.320
Base Mammoth	C2An.2r/C2An.3n	162.95	3.330
$\delta^{18}O$ stratigraphy		163.05	3.341
$\delta^{18}O$ stratigraphy		163.22	3.350
$\delta^{18}O$ stratigraphy		163.51	3.365
$\delta^{18}O$ stratigraphy		163.66	3.375
$\delta^{18}O$ stratigraphy		163.81	3.380
$\delta^{18}O$ stratigraphy		163.95	3.395
Top Gilbert	C2An.3n/C2Ar	168.83	3.596
HCO *Reticulofenestra pseudoumbilicus* (calcareous nannofossil)		185.80	3.820

SUPPLEMENTARY TABLE 8

Age model and stratigraphic events of Ocean Drilling Program (ODP) Hole 646B. The tie-points based on the palaeomagnetic reversals[24] and calcareous nannofossil stratigraphy[6] used to construct the age model for DSDP Hole 646B in ref. 21 are repeated here. The ages are reported according to the Geological Time Scale 2012[18]. Stratigraphic events in DSDP Hole 646B of *B. micropapillata* complex (HO at 323.40 mbsf), *C. devernaliae* (HO at 349.20 mbsf), *O.? eirikianum* (HO at 286.70 mbsf), *O. tegillatum* (HO at 325.12 mbsf), and *R. actinocoronata* (HO at 376.47 mbsf) are listed in Appendix A of ref. 2.

**Chron boundary name**<br>**Nannoplankton stratigraphy**	**Chron/Subchron**	**Depth** (mbsf)	**Age** (Ma)
Top Matuyama	C1n/C1r.1r	62.98	0.781
Jaramillo Top	C1r.1r/C1r.1n	76.70	0.988
Jaramillo Base	C1r.1n/C1r.2n	82.65	1.072
NN17/NN18 - NN19a		162.14	2.06
Top Gauss	C2r.2r/C2An.1n	191.50	2.581
Top Gilbert	C2An.3n/C2Ar	292.90	3.596
NN15 - NN16		343.02	3.79
NN13/NN14 - NN15		385.48	4.13
NN12 - NN13/NN14		455.29	5.13
NN11b - NN12		502.00	5.59
NN11a - NN11b		574.43	7.36
Chronozone C4, Top N2*		628.71	7.695
Chronozone C4, Base N2*		666.47	8.108

* Questionable identification of the palaeomagnetic reversal

Supplementary Note 1

At Iceland Sea ODP Hole 907A, prior to 4.5 Ma, the cool-tolerant taxa *Spiniferites elongatus* and *Impagidium pallidum* co-occur with thermophilic taxa suggesting temperate conditions. Although *S. elongatus* and *I. pallidum* can be considered as cool water species, both taxa have Northern Hemisphere distributions that reach as far south as 30°N and 45°N, respectively. *S. elongatus* comprises 1–10% of the total assemblage between 5 and 4.5 Ma at ODP Hole 907A. Such abundances are found today mainly in regions with sea surface temperature (SST) between –1 and 11 °C in winter and between 2 and 16 °C in summer, but can also occur in SSTs up to 25°C[25,26]. The other cool-water species *I. pallidum* is recorded occasionally in low abundances (1–2%) in the studied interval. In the modern and Pliocene oceans, such abundances occur in water temperatures mainly below 16°C, but have been recorded in SSTs up to ~25 °C[25–27]. The recorded abundances of *S. elongatus* and *I. pallidum* are thus not in conflict with temperate conditions for the Iceland Sea.
In contrast to the presence of cool-water taxa in temperate conditions, the diverse warm-water assemblage (including *Impagidinium aculeatum*, *Invertocysta lacrymosa*, *Melitasphaeridium choanophorum, Operculodinium israelianum, Operculodinium tegillatum*, and *Operculodinium*? *eirikianum*) at ODP Hole 907A has never been reported from (sub)polar waters and indicates temperate conditions in the Iceland Sea. For example, *M. choanophorum* is extinct in the mid- to high latitude North Atlantic since the late Pliocene[3] but was recently discovered in modern sediments of the northern Gulf of Mexico where summer SSTs are higher than 29°C[28]. Also, *O. israelianum* is a subtropical to tropical species that does not occur north of 50°N in the modern ocean[26]. The presence of these thermophilic taxa indicate the inflow of warm Atlantic waters at ODP Hole 907A, comparable to ODP Hole 642B.

SUPPLEMENTARY NOTE 2

The barren interval in Iceland Sea ODP Hole 907A between 4.14 and 2.6 Ma is only interrupted by three mono-specific *Spiniferites* sp. samples at 3.66 Ma, 3.42 Ma and 3.34 Ma. The paleoenvironmental significance of such assemblages is unclear, as the aberrant *Spiniferites* specimens with elaborate morphologies could not be determined to species level. Elaborate morphologies of *Spiniferites* could be a reflection of fresh water, possibly affecting the site via the East Greenland Current and/or melting sea ice and icebergs. The morphology–salinity relationship has been observed in the *Spiniferites cruciformis* complex from low-salinity environments[e.g.29-32].

SUPPLEMENTARY NOTE 3

Due to the warm temperate waters in the Norwegian Sea and the overall temperate conditions where tropical taxa can survive in the Iceland Sea between 5 and 4.5 Ma, a pronounced temperature gradient between the Iceland Sea and Norwegian Sea was absent. In fact, the dinoflagellate cyst records at ODP Hole 642B and ODP Hole 907A attest for a coupled temperature evolution of the Norwegian and Iceland Sea. Furthermore, a cooling recorded in the alkenone sea surface temperatures around 4.9–4.8 Ma (Supplementary Table 1) is accompanied by extremely high abundances of *Spiniferites elongatus* up to ~50% at ODP Hole 907A. Such abundances occur in the modern ocean only in Baffin Bay, where summer SSTs are around 5–6 °C. In Norwegian Sea ODP Hole 642B, an increase in the cool water species *Filisphaera filifera* causes a drop in the W/C index around this time (Figure 3), suggesting that the Iceland and Norwegian Sea temperature evolution are coupled. This Nordic Seas cooling may have been related to a global glaciation event (4.9–4.8 Ma) when glaciers expanded to the coastline in Greenland, Iceland and Scandinavia[33].

SUPPLEMENTARY NOTE 4

List with full authorial citation of the dinoflagellate cyst and acritarch taxa in the text, figures and supplementary notes. The dinoflagellate cyst nomenclature follows ref. 34, and full authorial citations can be found there. Authorial citations not listed in ref. 34 can be found in refs. 5, 21, 35–37.

Dinoflagellate cysts

Achomosphaera Evitt 1963

Achomosphaera andalousiensis subspecies *suttonensis* Head 1997

Ataxiodinium? sp. A (this study)

Batiacasphaera micropapillata complex (Schreck and Matthiessen 2013)

  This complex comprises *Batiacasphaera micropapillata* Stover 1977 and *Batiacasphaera minuta* (Matsuoka 1983) Matsuoka and Head 1992

Bitectatodinium tepikiense Wilson 1973

Corrudinium devernaliae Head and Norris 2003

Cysts of *Protoceratium reticulatum* (Claparède and Lachmann, 1859) Bütschli, 1885

  Note: the name cysts of *Protoceratium reticulatum* was recently proposed for *Operculodinium centrocarpum* sensu Wall and Dale (1966) in ref. 36

Dapsilidinium pseudocolligerum (Stover 1977) Bujak et al. 1980

Filisphaera filifera Bujak (1984) Head 1994

Habibacysta tectata Head et al. 1989

Hystrichokolpoma rigaudiae Deflandre and Cookson 1955

Impagidinium Stover and Evitt 1978

Impagidinium aculeatum (Wall 1967) Lentin and Williams 1981

Impagidinium cf. *pacificum* Bujak 1984

Impagidinium pallidum Bujak 1984

Impagidinium paradoxum (Wall 1967) Stover and Evitt 1978

Impagidinium patulum (Wall 1967) Stover and Evitt 1978

Impagidinium solidum Versteegh and Zevenboom 1995

Invertocysta Edwards 1984

Invertocysta lacrymosa Edwards 1984

Invertocysta tabulata Edwards 1984

Lingulodinium machaerophorum (Deflandre and Cookson 1955) Wall 1967

Melitasphaeridium choanophorum (Deflandre and Cookson 1955) Harland and Hill 1979

Nematosphaeropsis labyrinthus (Ostenfeld 1903) Reid 1974

Operculodinium centrocarpum s.s. (Deflandre and Cookson 1955) Wall 1967

Operculodinium? *eirikianum* (Head et al. 1989) Head 1997

Operculodinium israelianum (Rossignol 1982) Wall 1967

Operculodinium tegillatum Head 1997

Pyxidinopsis vesiculata Head and Norris 2003

Reticulatosphaera actinocoronata (Benedek 1972) Bujak and Matsuoka 1986

Protoperidinioids

Spiniferites (Mantell 19850) Sarjeant 1970

Spiniferites elongatus Reid 1974

Spiniferites mirabilis (Rossignol 1964) Sarjeant 1970

Spiniferites hyperacanthus (Deflandre and Cookson 1955) Cookson and Eisenack 1974

Tectatodinium pellitum (Wall 1967) Head 1994

Tuberculodinium vancampoae Wall 1967

Acritarchs

Cymatiosphaera? *invaginata* Head, Norris and Mudie 1989

Cyst type I of de Vernal and Mudie (1989)

Lavradosphaera crista De Schepper and Head 2008

SUPPLEMENTARY REFERENCES

1. Schreck, M., Meheust, M., Stein, R. & Matthiessen, J. Response of marine palynomorphs to Neogene climate cooling in the Iceland Sea (ODP Hole 907A). *Mar. Micropaleontol.* **101,** 49–67 (2013).
2. Schreck, M., Matthiessen, J. & Head, M. J. A magnetostratigraphic calibration of Middle Miocene through Pliocene dinoflagellate cyst and acritarch events in the Iceland Sea (Ocean Drilling Program Hole 907A). *Rev. Palaebot. Palynol.* **187,** 66–94 (2012).
3. De Schepper, S. & Head, M. J. Pliocene and Pleistocene dinoflagellate cyst and acritarch zonation of DSDP Hole 610A, eastern North Atlantic. *Palynology* **33,** 179–218 (2009).
4. De Schepper, S. & Head, M. J. Age calibration of dinoflagellate cyst and acritarch events in the Pliocene–Pleistocene of the eastern North Atlantic (DSDP Hole 610A). *Stratigraphy* **5,** 137–161 (2008).
5. de Vernal, A. & Mudie, P. Pliocene and Pleistocene palynostratigraphy at ODP Sites 646 and 647, eastern and southern Labrador Sea. *Proc. ODP Sci. Res.* **105,** 401–422 (1989).
6. Knüttel, S., Russell, M. D. J. & Firth, J. V. Neogene Calcareous Nannofossils From ODP Leg 105: Implications For Pleistocene Paleoceanographic Trends. *Proc. ODP Sci. Res.* **105,** 245–262 (1989).
7. Head, M. J. & Norris, G. New species of dinoflagellate cysts and other palynomorphs from the latest Miocene and Pliocene of DSDP Hole 603C, western North Atlantic. *J. Paleontol.* **77,** 1–15 (2003).
8. Canninga, G. & Zijderveld, J. Late Cenozoic magnetostratigraphy of deep Sea Drilling Project Hole 603C, Leg 93, on the North American continental rise off Cape Hatteras. *DSDP Init. Rep.* **93,** 839–848 (1987).
9. De Schepper, S., Head, M. J. & Louwye, S. Pliocene dinoflagellate cyst stratigraphy, palaeoecology and sequence stratigraphy of the Tunnel-Canal Dock, Belgium. *Geol. Mag.* **146,** 92–112 (2009).
10. Meijer, T. Stratigraphical notes on *Macoma* (Bivalvia) in the southern part of the North Sea Basin and some remarks on the arrival of Pacific species. *Scripta Geologica Spec. Issue* **2,** 297–312 (1993).
11. Marquet, R. The Neogene Bivalvia (Heterodonta and Anomalodesmata) and Scaphopoda from Kallo and Doel (Ooest-Vlaanderen, Belgium). *Paleontos* **6,** 1–142 (2006).
12. Head, M. J. Thermophilic dinoflagellate assemblages from the mid Pliocene of eastern England. *J. Paleontol.* **71,** 165–193 (1997).
13. Long, P. E. & Zalasiewicz, J. A. The molluscan fauna of the Coralline Crag (Pliocene, Zanclean) at Raydon Hall, Suffolk, UK: Palaeoecological significance reassessed. *Palaeogeogr. Palaeoclimatol. Palaeoecol.* **309,** 53–72 (2011).
14. Wijnker, E. *et al.* Neogene stratigraphy of the Langenboom locality (Noord-Brabant, the Netherlands). *Neth. J. Geosci.* **87,** 165–180 (2008).
15. Verhoeven, K., Louwye, S., Eiríksson, J. & De Schepper, S. A new age model for the Pliocene-Pleistocene Tjörnes section on Iceland: Its implication for the timing of North Atlantic-Pacific palaeoceanographic pathways. *Palaeogeogr. Palaeoclimatol. Palaeoecol.* **309,** 33–52 (2011).
16. Durham, J. W. & MacNeil, F. S. Cenozoic migrations of marine invertebrates through the Bering Strait region, in *The Bering Land Bridge* (Hopkins, D. M.) 326–349 (Stanford University Press, 1967).
17. Bleil, U. Magnetostratigraphy of Neogene and Quaternary sediments series from the Norwegian Sea: Ocean Drilling Program, Leg 104. *Proc. ODP Sci. Res.* **104,** 829–901 (1989).
18. Hilgen, F. J. *et al.* The Neogene Period, in *The Geologic Time Scale 2012* (Gradstein, F., Ogg, J. G., Schmitz, M. & Ogg, G.) 923–978 (Elsevier, 2012).
19. Channell, J., Amigo, A., Fronval, T., Rack, F. & Lehman, B. Magnetic stratigraphy at Sites 907 and 985 in the Norwegian-Greenland Sea and a revision of the Site 907 composite section. *Proc. ODP Sci. Res.* **162,** 131–148 (1999).
20. Lourens, L. J., Hilgen, F. J., Laskar, J., Shackleton, N. J. & Wilson, D. in *A Geological Time Scale 2004* (Gradstein, F., Ogg, J. G. & Smith, A.) 409–430 (A Geological Time Scale 2004, 2005).
21. De Schepper, S. & Head, M. J. New late Cenozoic acritarchs: evolution, palaeoecology and correlation potential in high latitude oceans. *J. Syst. Paleont.* **12,** 493–519 (2014).

22. Clement, B. & Robinson, F. The magnetostratigraphy of Leg 94 sediments. *DSDP Init. Rep.* **94,** 635–650 (1987).
23. Kleiven, H., Jansen, E., Fronval, T. & Smith, T. Intensification of Northern Hemisphere glaciations in the circum Atlantic region (3.5–2.4 Ma) — ice-rafted detritus evidence. *Palaeogeogr. Palaeoclimatol. Palaeoecol.* **184,** 213–223 (2002).
24. Clement, B. M., Hall, F. J. & Jarrard, R. D. The magnetostratigraphy of Ocean Drilling Program Leg 105 sediments. *Proc. ODP Sci. Res.* **105,** 583–595 (1989).
25. Marret, F. & Zonneveld, K. A. F. Atlas of modern organic-walled dinoflagellate cyst distribution. *Rev. Palaebot. Palynol.* **125,** 1–200 (2003).
26. Zonneveld, K. A. F. *et al.* Atlas of modern dinoflagellate cyst distribution based on 2405 datapoints. *Rev. Palaebot. Palynol.* **191**, 1–197 (2013).
27. De Schepper, S., Fischer, E., Groeneveld, J., Head, M. J. & Matthiessen, J. Deciphering the palaeoecology of Late Pliocene and Early Pleistocene dinoflagellate cysts. *Palaeogeogr. Palaeoclimatol. Palaeoecol.* **309,** 17–32 (2011).
28. Limoges, A., Londeix, L. & de Vernal, A. Organic-walled dinoflagellate cyst distribution in the Gulf of Mexico. *Mar. Micropaleontol.* **102,** 51–68 (2013).
29. Ellegaard, M. Variations in dinoflagellate cyst morphology under conditions of changing salinity during the last 2000 years in the Limfjord, Denmark. *Rev. Palaebot. Palynol.* **109,** 65–81 (2000).
30. Mudie, P. J., Rochon, A., Aksu, A. E. & Gillespie, H. Dinoflagellate cysts, freshwater algae and fungal spores as salinity indicators in Late Quaternary cores from Marmara and Black seas. *Mar. Geol.* **190,** 203–231 (2002).
31. Marret, F., Leroy, S. A. G., Chalié, F., and Gasse, F. New organic-walled dinoflagellate cysts from recent sediments of Central Asian seas. *Rev. Palaebot. Palynol.* 129, 1–20 (2004).
32. Sorrel, P. *et al.* Hydrographic development of the Aral Sea during the last 2000 years based on a quantitative analysis of dinoflagellate cysts. *Palaeogeogr. Palaeoclimatol. Palaeoecol.* **234,** 304–327 (2006).
33. De Schepper, S., Gibbard, P. L., Salzmann, U. & Ehlers, J. A global synthesis of the marine and terrestrial evidence for glaciation during the Pliocene Epoch. *Earth Sci. Rev.* **135,** 83–102 (2014).
34. Fensome, R. A., MacRae, R. A. & Williams, G. L. Dinoflaj2, version 1. *AASP Data Series* **1,** (2008).
35. Schreck, M. & Matthiessen, J. *Batiacasphaera micropapillata*: palaeobiogeographic distribution and palaeoecological implications of a critical Neogene species complex, in *Biological and Geological Perspectives of Dinoflagellates* (Lewis, J. M., Marret, F. & Bradley, L.) 293–306 (The Micropalaeontological Society, Special Publications, 2013).
36. Paez-Reyes, M. & Head, M. J. The Cenozoic gonyaulacacean dinoflagellate genera *Operculodinium* Wall, 1967 and *Protoceratium* Bergh, 1881 and their phylogenetic relationships. *J. Paleontol.* **87,** 786–803 (2013).
37. Head, M.J., Norris, G., & Mudie, P.J. New species of dinocysts and a new species of acritarch from the Upper Miocene and lowermost Pliocene, ODP Leg 105, Site 646, Labrador Sea. *Proc. ODP Sci. Res.* **105,** 453–466 (1989).
